# Paraformaldehyde-Induced Vesiculation Preserves Native Membrane Physiological States Critical for T‑Cell Activation

**DOI:** 10.1021/acsomega.6c04767

**Published:** 2026-09-03

**Authors:** Dilip Shrestha, Veerawut Veerapongchai, Christian Eggeling

**Affiliations:** † MRC Human Immunology Unit, Weatherall Institute of Molecular Medicine, University of Oxford, Oxford OX3 9DS, U.K.

## Abstract

Plasma membrane vesicles (PMVs) are powerful model systems for studying plasma membrane biophysics, but their production typically relies on chemicals, such as dithiothreitol (DTT) and paraformaldehyde (PFA). DTT is detrimental to protein structure and can alter membrane composition, whereas PFA is a well-established fixative that is widely used in biological studies. Taking this into consideration, we introduced a DTT-free method for generating PMVs using PFA alone. Using flow cytometry and fluorescence microscopy, we validated its robustness across multiple cell types. Measurements with the environment-sensitive probe C-Laurdan show that PMVs produced with PFA plus DTT display higher lipid packing compared to those generated with PFA alone, indicating an effect of DTT on membrane order. Furthermore, fluorescence correlation spectroscopy (FCS) measurements reveal reduced mobility of the immune receptor cluster of differentiation 1d (CD1d) on DTT-treated PMVs. During the evaluation of PMVs in coculture experiments, we further identified residual PFA as the source of cellular toxicity, which was successfully eliminated by dialysis. Finally, we demonstrate the biological applicability of the resulting PMVs by assessing T-cell activation, showing that they preserve key physiological properties of the source cells. Overall, our findings suggest that PMVs generated using PFA alone more faithfully preserve the native properties of the source cell plasma membrane than those produced with the conventional PFA plus DTT protocol. Combined with the effective removal of residual PFA by dialysis, this approach expands the potential of PMVs as physiologically relevant model systems for biological and biomedical research.

## Graphical abstract

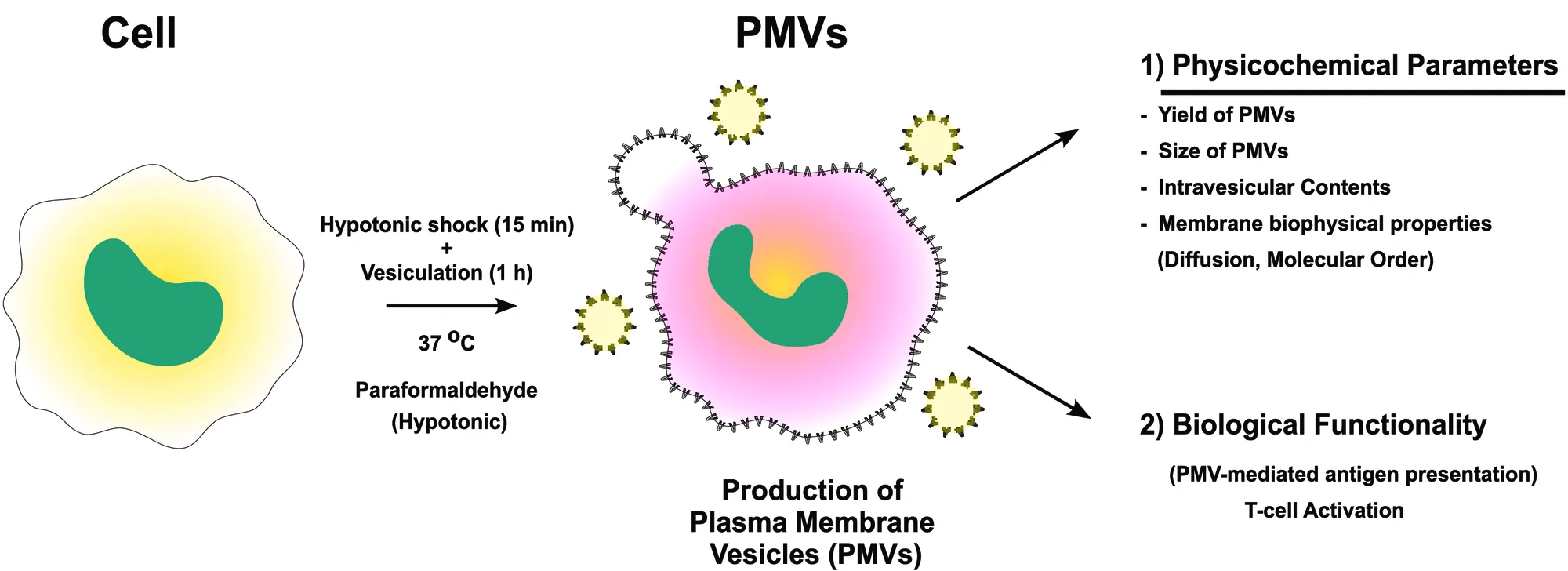

## 1 Introduction

The plasma membrane (PM) and the organization of its lipids and proteins play a central role in regulating the molecular processes involved in cellular signaling. Distinct biophysical features such as lipid order and packing are now recognized as critical determinants of numerous physiological functions.^1–3^ Cell membranes exhibit coexisting liquid-ordered (Lo) and liquid-disordered (Ld) regions, characterized by tightly and loosely packed lipids, respectively. These variations in lipid ordering give rise to local differences in molecular mobility, organization, and interaction dynamics. Consequently, direct investigation of these membrane features in living cells remains challenging because of their dynamic molecular heterogeneity and nanoscale organization.^4^ Plasma membrane vesicles (PMVs) have therefore emerged as a powerful tool for investigating these functional membrane properties, and their discovery has been instrumental in establishing the concept of Lo and Ld domains in the PM.^5^

Historically, PMVs are generated via chemicals, for example using dithiothreitol (DTT) and paraformaldehyde (PFA), or *N*-ethylmaleimide (NEM). PFA is typically used in combination with DTT, whereas NEM can act alone.^5–8^ We recently reported physicochemical differences in the membrane of PMVs produced using these reagents,^9^ and earlier work also documented disparities in their membrane biophysical^7,10–12^ and likely their biochemical properties.^12^ Hence, there are reasonable concerns about the PFA plus DTT method. PFA is applied at a relatively low concentration (0.1%, ∼25 mM), far below those used for fixation or molecular immobilization, and this concentration has minimal impact on membrane stiffness when compared to the live cell PM.^13,14^ However, DTT is a strong reducing agent and therefore can modify proteins by disrupting disulfide bonds.^15,16^ Thus, a DTT-free strategy relying solely on PFA would be advantageous for preserving native protein function.

Few studies have demonstrated the feasibility of generating PMVs using PFA alone.^17,18^ These protocols required prolonged incubation times (8–24h), and biochemical analyses revealed enrichment of cytosolic proteins and degraded RNA within the resulting vesicles.^17,19^ While these findings provided valuable insights, PMVs were not subjected to comprehensive biophysical characterization, leaving unresolved whether PFA-induced vesicles faithfully reproduce the physicochemical properties of the PM in living cells. Here, it is important to note that PMV production depends on membrane blebbing from living cells, and aldehyde-induced blebbing is a well-established phenomenon in microscopy. Typically, membrane blebbing occurs within ∼1 h of aldehyde exposure,^20,21^ which raises the concern that the extended incubation time used in previous PFA-only approaches may promote changes in the membrane composition, alter intravesicular contents, and likely increase PMV heterogeneity. Furthermore, adherent cell lines, such as CHO and RBL cells, are generally preferred for PMV generation because they efficiently produce PMVs that readily detach into the surrounding medium,^5,6,11,22^ In contrast, suspension cells, such as Jurkat T cells, often yield fewer PMVs under standard protocols,^22^ limiting the applicability of existing methods.

To address these issues, we aimed to develop a PFA-based protocol producing PMVs on a time scale comparable to conventional PFA plus DTT method,^5,23^ while avoiding the potential artifacts introduced by DTT. We tested the effect of brief exposure to hypotonic buffer on the vesicle yield from suspension cells and found that it substantially enhances PMV production. We then systematically characterized the resulting vesicles using flow cytometry to quantify yield, size distribution, and intravesicular content,^9^ as well as advanced fluorescence microscopy to assess membrane fluidity and molecular diffusion.^5,9^ Our results showed that PFA alone can generate PMVs across diverse cell types, with yields markedly increased by a brief hypotonic buffer treatment. Given the adverse effects of DTT on protein structures, we also investigated its influence on the local molecular environment of the PM, particularly lipid packing and hydration. We evaluated membrane packing and measured the diffusion of the immune receptor cluster of differentiation 1d (CD1d), known for its role in modulating immune responses.^24^ Lipid packing was assessed via generalized polarization (GP) measurements using the environment-sensitive probe C-Laurdan^25^ and the mobility of CD1d was determined using a single-molecule-sensitive technique fluorescence correlation spectroscopy (FCS)^9^ on the surface of PMVs. Both approaches revealed clear biophysical differences between PMVs generated using our new PFA-only protocol and those produced by the conventional PFA plus DTT method.

PMVs have become well-established model systems for investigating the PM organization and dynamics. However, their applications in biological studies have remained limited. Therefore, one of the major objectives of this study was to evaluate the suitability of PMVs for biological applications. During the course of this work, we observed that residual PFA in the PMV suspension after vesicle production exerted cytotoxic effects in coculture experiments. This prompted us to develop a strategy for eliminating residual PFA and, thereby, improving the biocompatibility of the PMV preparations. Using this optimized protocol, we subsequently demonstrated the suitability of PFA-only PMVs for biological applications in T-cell activation experiments. In particular, our results show that these PMVs preserve key physiological properties of the source cells, exemplified here by functional antigen presentation.

In summary, we present a robust and rapid DTT-free method for generating PMVs that avoids the detrimental effects of chemicals while maintaining cytocompatibility. This approach enhances the fidelity of PMV-based studies and offers substantial advantages over the traditional PFA plus DTT protocol, paving the way for broader adoption of PMVs in membrane biology research and beyond.

## 2 Materials and Methods

### 2.1 Cell Culture

The following cell lines were used in this study: Jurkat T cells, HeLa cells, THP-1 CD1d cells, and Jurkat invariant natural killer T (Jurkat iNKT) cells. Jurkat and HeLa cells were obtained from ATCC. Jurkat iNKT cells were provided by Prof. Peter Steinberger (University of Vienna, Austria). The biological characteristics of this cell line have been described previously by Humeniuk et al.^26^ All cell lines were cultured in a RPMI medium supplemented with 10% fetal calf serum, 5 mM l-glutamine (Sigma-Aldrich, UK), and 1% penicillin–streptomycin (Sigma-Aldrich, UK). Cells were maintained at a density of 1–3 × 10^6^ cells/mL in standard culture flasks at 37 °C and 5% CO_2_. HeLa cells were passaged upon reaching confluence, typically every 2 days.

### 2.2 Buffers and Chemicals

Two types of vesiculation buffers differing in sodium chloride (NaCl) concentration were used in this study. Both buffers contained 10 mM HEPES, 2 mM calcium chloride (CaCl_2_), and either 150 mM NaCl or 50 mM NaCl (all reagents from Sigma-Aldrich, UK). pH of the buffer was adjusted to 7.4. Hereon, buffers containing 150 mM or 50 mM NaCl are referred to as isotonic and hypotonic vesiculation buffers, respectively. DTT stock solutions were prepared in water, aliquoted, and stored at −20 °C. Cell-permeable DNA/RNA-binding dye, acridine orange (AO), and 16% aqueous PFA solution were purchased from Fisher Scientific UK. Annexin V-FITC and propidium iodide (PI), used for live/dead assays, were purchased from BioLegend (UK).

### 2.3 Production of PMVs

PMVs were generated either using the previously described PFA plus DTT protocol in isotonic buffer^5^ or using a modified protocol involving a hypotonic shock step. Briefly, 2 × 10^6^ cells were collected in 1.5 mL tubes and washed twice in isotonic buffer by centrifuging at 1600 rpm for 3 min. After the second wash, cells were transferred to a 6-well plate and incubated in either isotonic or hypotonic buffer for 15 min at 37 °C, as detailed in Table 1. Where required, the buffer was then gently removed and replaced with 1 mL isotonic buffer containing the vesiculating agents (either PFA plus DTT or PFA alone). Cells were incubated for an additional 1 h at 37 °C to induce vesiculation. Following incubation, the plate was placed on a shaker (20 rpm) for 20 min to promote vesicle detachment. PMVs were then collected from the supernatant for downstream analysis. For Jurkat T cells, implementation of this protocol was straightforward. Adherent cell line, HeLa cells, however required seeding at 3 × 10^5^ cells per well in a 6-well plate overnight. The following day, washing, osmotic shock, and vesiculation steps were performed as described above.

**Table 1:** Incubation and Buffer Conditions^a^

Conditions	Osmotic shock (15 min)	Vesiculation (1 h)
1. Control (Untreated)	hypotonic buffer	
2. PFA plus DTT (Isotonic)	isotonic buffer	isotonic buffer
3. PFA plus DTT (Hypotonic)	hypotonic buffer	isotonic buffer
4. PFA (Isotonic)	isotonic buffer	isotonic buffer
5. PFA (Hypotonic)	hypotonic buffer	isotonic buffer
6. PFA (Unchanged Hypotonic)	hypotonic buffer	hypotonic buffer

a The first incubation for 15 min was an osmotic-shock step. Vesiculation was performed either with PFA alone or PFA plus DTT containing buffers for 1 h, which are mentioned in the first and third columns.

### 2.4 Flow Cytometry of Vesicles and Cells

Flow cytometric measurements of vesicles and cells were performed using an Invitrogen Attune NxT flow cytometer (Thermo Fisher Scientific, UK). Forward scatter (FSC) and side scatter (SSC) voltage thresholds were empirically determined ensuring exclusion of background noise arising from buffer scattering and cellular debris. Filtered PBS (0.2 μm) and Apogee calibration beads were used to calibrate the instrument. Apogee beads have a refractive index of 1.42, comparable to that of biological vesicles, and therefore are an appropriate standard for PMVs in our experimental setup. For each sample, 50 μL of sample volume was acquired at a flow rate of 25 μL/min. PMVs and intact cells were readily distinguishable based on their scattering properties. In addition, AO was used to enhance detection and improve discrimination between PMVs and cells.

### 2.5 Dialysis of PMVs

To remove residual PFA, PMVs were dialyzed immediately after vesiculation using a 50 mL Slide-A-Lyzer MINI Dialysis Devices (10 kDa MWCO; Thermo Fisher Scientific, UK). Dialysis membranes were prewetted in water prior to use. Briefly, PMVs were generated in 6-well plates as described above and centrifuged at 1600 rpm for 3 min to remove intact cells and large debris. The supernatant containing PMVs was then transferred into the dialysis device. The outer tube was filled with 43 mL isotonic buffer, and dialysis was performed on a shaker at 300 rpm for 3 h. The buffer was then replaced with fresh isotonic buffer (43 mL), and dialysis was continued for an additional 21 h. Next day, samples were collected for downstream analysis. Aliquots (∼300 μL) were also collected at time-points 0 h, i.e., no dialysis, and after 3 h of dialysis, and stored at −20 °C for subsequent experiments.

### 2.6 Live–Dead Assay for Cell Viability

Cell viability experiments were performed using Jurkat iNKT cells. Briefly, 1 × 10^6^ Jurkat iNKT cells were coincubated with PMV preparations obtained after 0, 3, or 24 h of dialysis in a 48-well plate. Cells were incubated for 24 h at 37 °C. The ratio of culture medium to PMV-containing buffer was maintained at 3:1. Following incubation, cells were centrifuged twice in isotonic buffer at 1600 rpm for 3 min. The resulting pellet was then resuspended in 300 μL isotonic buffer and stained with Annexin V-FITC (1 μL) and PI (1 μL). Samples were incubated in the dark for 15 min at room temperature (RT). Viability was then quantified using the Attune NxT flow cytometer. FITC and PI were excited using the 488 and 561 nm lasers, respectively, and emitted fluorescence signals were collected using the BL1 (530/30) and YL1 (585/16) detectors. Data were analyzed using FlowJo v10.

### 2.7 Antigen Presentation Assay Using PMV–Jurkat iNKT Cell Coculture

Numerous studies have used THP1-CD1d as antigen-presenting cells (APCs) for iNKT cells in antigen presentation experiments.^24,27^ Hence, we selected THP1-CD1d cells for generating PMVs. Two conditions were selected to represent distinct physiological states: (i) nonstimulatory control condition, i.e., cultured without the lipid antigen α-galactosylceramide (α-GalCer, buffer only condition) and (ii) stimulatory condition, i.e., cultured in the presence of α-GalCer for 22 h (antigen-pulsed). Next day, PMVs were generated as described above. To remove residual cells (>10 μm in size), PMV preparations were passed through pluriStrainer (Cambridge Biosciences, UK) with a pore size of 5 μm, resulting in highly pure vesicles. PMVs were then dialyzed for 24 h and subsequently quantified using the Attune NxT flow cytometer. For coculture experiments, Jurkat iNKT cells (1 × 10^6^) were incubated with PMVs, at PMVs-to-cell ratios of 1:1 and 2:1, in a 48-well plate for 24 h. After incubation, Jurkat iNKT cells were collected, washed, and stained for CD69—a marker for T-cell activation. Subsequently, samples were analyzed by flow cytometry for the level of expression of CD69 and NF-κB-eGFP proteins. Data were analyzed using FlowJo v10.

### 2.8 Assessment of Membrane Fluidity: GP Imaging

Membrane fluidity was determined by GP measurements using C-Laurdan following an established protocol.^25^ PMVs were labeled with C-Laurdan (∼0.5 μM) for 2 min at RT then transferred to ibidi glass-bottom chambers (#1.5) for imaging. Spectral imaging was performed using a Zeiss LSM 880 confocal microscope equipped with an oil-immersion Plan-Apochromat 63×/1.4 NA objective. C-Laurdan was excited with a 405 nm laser and fluorescence emission was collected using a 32-channel GaAsP detector after dispersion through a diffraction grating mechanism. Spectral data were acquired at 8.9 nm resolution over the range of 415–695 nm. Spectral images were analyzed using a GP plugin.^25^ Emission spectra were fitted with a Gaussian function, and intensity values at the relevant wavelengths were extracted from the fits. GP values were calculated according to eq 1 using the fluorescence intensities measured at wavelength characteristic of the Lo and Ld membrane phases.

1 $$GP=\frac{I_{Lo}-I_{Ld}}{I_{Lo}+I_{Ld}}$$

where “*I*_Lo_” and “*I*_Ld_” denote the fluorescence intensities measured at 440 nm (λ_Lo_) and 495 nm (λ_Ld_), respectively. These wavelengths were selected because they provide high sensitivity to changes in the membrane order as reflected by the GP value.

### 2.9 Measuring Lateral Diffusion of CD1d with FCS

Mobility of the membrane-embedded CD1d receptors was quantified using FCS. Experiments were performed on a Zeiss LSM 880 inverted confocal microscope equipped with a 40× C-apochromat water-immersion objective (NA 1.2, W Corr FCS; Zeiss). CD1d on the surface of PMVs was labeled with Alexa Fluor 488-conjugated Fab antibody fragments by incubating the sample for 1 h at RT (final concentration, 10 μg/mL; clone 51.1.3). Labeled PMVs were subsequently transferred to an 8 well μ-Slide glass-bottom ibidi chamber. Alexa Fluor 488 was excited using a 488 nm laser, and fluorescence emission was collected between 500 and 600 nm. The experimental confocal volume was calibrated using the known diffusion coefficient (“*D*”) of Alexa Fluor 488 in water at 25 °C (*D* = 414 μm^2^/s). Measurements were performed on the top membrane of PMVs. Autocorrelation curves generated from the Zeiss instrument were fitted using FoCuS-point software, applying a one-component 2-D diffusion model and a correction for triplet-state.^28^ “*D*” values were subsequently determined using eq 2 from measurements performed on at least 15 PMVs for each experimental group.

2 $$D=\frac{\omega^{2}}{8.\ln\left(2\right).\tau_{D}}$$

where “ω” represents the full width at half-maximum (FWHM) of the confocal detection volume and “τ_*D*_” is the mean transit time of fluorescent molecules through the confocal spot. “τ_*D*_” was obtained directly from fitted autocorrelation curves, while “ω” was determined from calibration measurements using the Alexa Fluor 488 dye.

### 2.10 Data Analysis

Graphs and statistical analyses were performed using GraphPad Prism (version 10.0). Appropriate statistical tests used in the experiments are indicated in the corresponding figure legends. Unless explicitly stated, the data are presented as mean ± standard deviation (SD).

## 3 Results

### 3.1 Optimization of a PFA-Based Method for the Generation of PMVs

PFA is commonly used in combination with DTT to generate the PMVs. However, the toxicity of DTT and its propensity to denature proteins raise concerns regarding its impact on the molecular composition of the PM. To address these limitations, we sought to develop a protocol capable of producing PMVs using PFA alone, without DTT, from both suspension and adherent cells.

To facilitate efficient vesiculation in the absence of DTT, we explored the use of hypotonic condition, which can promote membrane blebbing by disrupting the link between the membrane and cortical actin cytoskeleton.^29^ Six experimental conditions were tested, as detailed in Table 1, using THP-1 CD1d cells. Vesicles were identified using the nucleic acid binding dye AO, which enabled clear discrimination between scattering from the buffer and PMVs in flow cytometry (Figure S1). Compared to control condition, which contained buffer instead of chemical, all other treatments, either using PFA or DTT or both reagents, resulted in enhanced PMV production (Figure 1C). FSC, which correlates with vesicle size, increased under all chemical conditions (Figure 1A). In contrast, SSC was reduced under all conditions relative to the control, suggesting that PMVs generated by chemical blebbing incorporate less intracellular material and cellular debris (Figure 1B). The mean ± SD concentration was 1.04 ± 0.25 × 10^6^ PMVs/mL for the conventional PFA plus DTT (Isotonic) method, compared with 0.11 ± 0.007 × 10^6^ PMVs/mL for the control condition. The highest vesicle yield was observed with the conventional PFA plus DTT (Isotonic) method, showing an average ∼9.6-fold increase over control. Importantly, all PFA-only conditions also yielded substantially higher PMV concentrations than the control, irrespective of the osmotic conditions. Among these, the PFA (Hypotonic) protocol produced the highest PMV yield (0.78 ± 0.12 × 10^6^ PMVs/mL), corresponding to an approximately 7-fold increase over the control (Figure 1). Surprisingly, incorporating a hypotonic shock step into the conventional PFA plus DTT method resulted in the lowest vesicle yield among all tested conditions (Figure 1C). Collectively, these findings demonstrate that PFA alone, when combined with hypotonic treatment, is an effective strategy for inducing PMVs.

**Figure 1:**
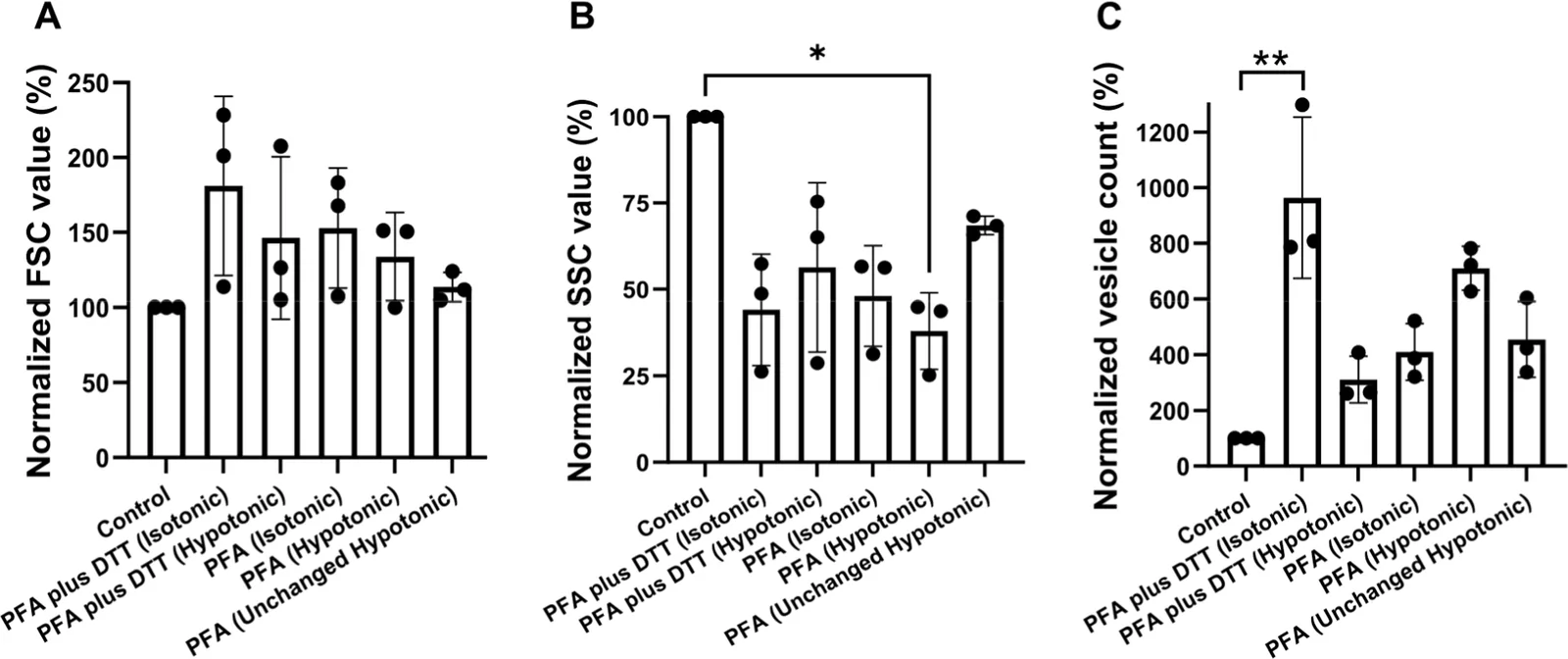
Optimization and comparison of methods for producing PMVs. PMVs were generated from THP1-CD1d cells employing various methods listed in Table 1 and compared for their (A) size (FSC); (B) intravesicular contents (SSC); and (C) yield (total PMV output). Data were normalized to the values from the control (buffer only condition), which was defined as 100% for each day. Each symbol represents an independent experimental replicate performed on a separate day, *n* = 3. Statistical significance was determined using a Kruskall–Wallis test and Dunn’s post hoc multiple-comparison test. Significant differences are displayed as “*” and “**” indicating *p*-values < 0.05 and <0.01, respectively; non- significant *p*-values are not shown in the figure.

### 3.2 The PFA (Hypotonic) Method Is Efficient at Generating PMVs from Both Adherent and Suspension Cells

Having established the PFA (Hypotonic) method, we next evaluated its applicability across different cell types, including adherent HeLa cells and vesiculation-resistant Jurkat T cells. For this comparison, we focused primarily on two conditions: PFA (Hypotonic) and the conventional PFA plus DTT (Isotonic) protocol. Consistent with the observations in THP-1 CD1d cells, the PFA (Hypotonic) method was equally effective at inducing PMVs from both cell types (Figure 2). In Jurkat T cells, the PFA (Hypotonic) protocol yielded significantly higher PMV concentration than the conventional PFA plus DTT (Isotonic) method (Figure 2C), whereas no significant difference was observed between the two protocols in HeLa cells (Figure 2F). The numbers of PMVs generated per cell were 1.37 ± 0.24 and 1.79 ± 0.55 for the PFA plus DTT (Isotonic) and PFA (Hypotonic) methods, respectively, in Jurkat T cells, and 1.86 ± 0.5 and 1.79 ± 0.38, respectively, in HeLa cells. Consistently, the corresponding PMV concentrations (mean ± SD) were 2.75 ± 0.48 × 10^6^ and 3.57 ± 1.1 × 10^6^ PMVs/mL for Jurkat T cells and 0.56 ± 0.15 × 10^6^ and 0.54 ± 0.12 × 10^6^ PMVs/mL for HeLa cells. It should be noted that different initial cell numbers were used for PMV generation from Jurkat and HeLa cells. Analysis of FSC values revealed a general trend toward larger vesicles with the PFA plus DTT (Isotonic) method for both Jurkat and HeLa cells, reaching statistical significance only in HeLa cells. In contrast, vesicles generated under PFA (Hypotonic) conditions did not differ significantly in size from control vesicles. Consistent with the previous observation, SSC values were significantly lower for vesicles generated by both methods compared to that from controls, indicating reduced intravesicular content (Figure 2B,E). These results thus establish a PFA (Hypotonic) approach as a broadly applicable method for generating PMVs from both adherent and suspension cells.

**Figure 2:**
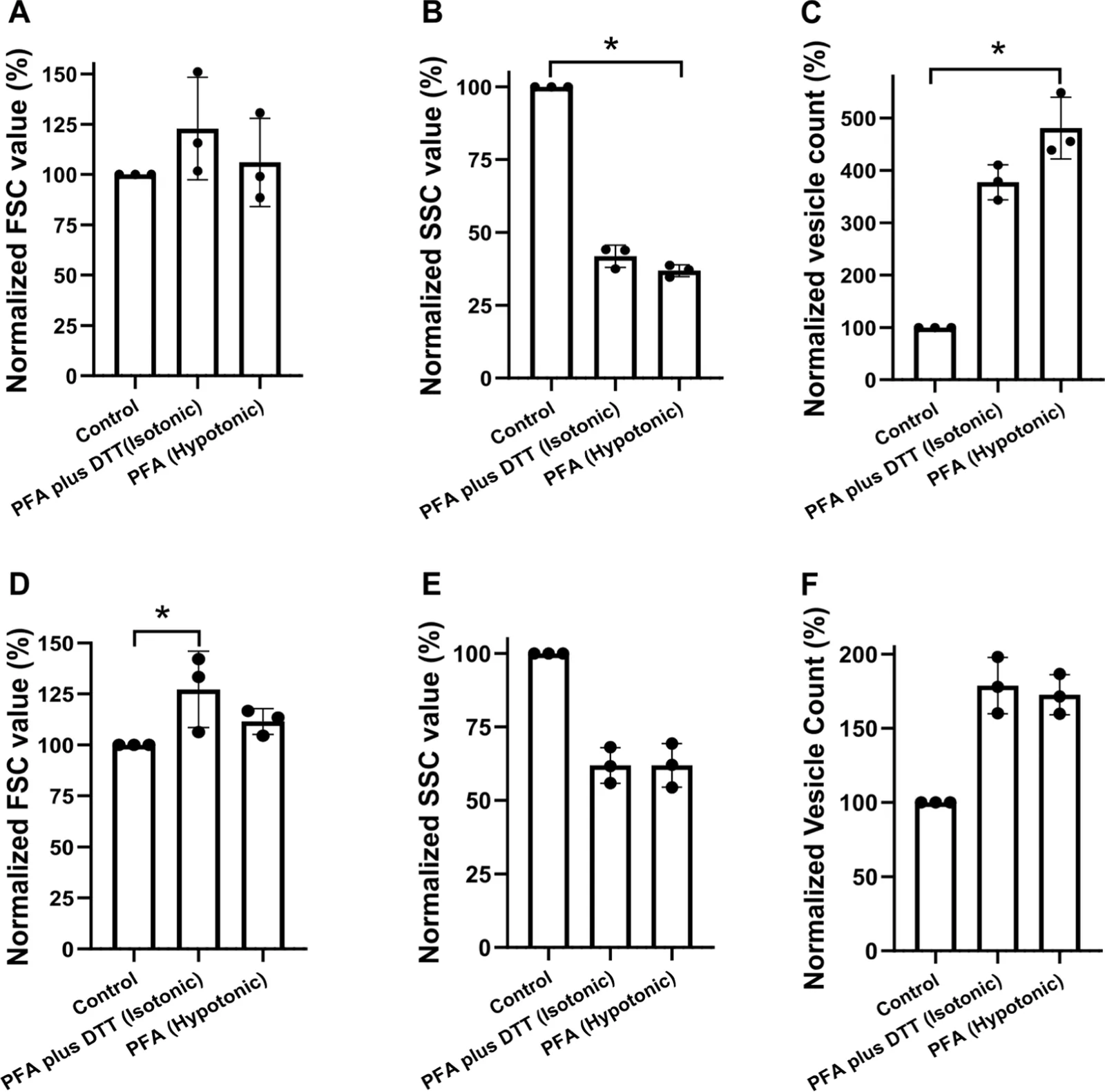
Yield and features of PMVs isolated using the PFA (Hypotonic) method from adherent and blebbing-resistant cell lines. HeLa and Jurkat T cell lines were used to generate PMVs using PFA plus DTT (Isotonic) and PFA (Hypotonic) methods. Various attributes of the PMVs were then compared: Jurkat T cell (A) FSC, (B) SSC and (C) Yield; and HeLa cell (D) FSC, (E) SSC, and (F) Yield. Data were normalized as described earlier in Figure 1 considering the values from control as 100%. Each symbol represents an independent experimental replicate performed on a separate day, *n* = 3. Statistical significance was determined using a Kruskall–Wallis test and Dunn’s post hoc multiple-comparison test. *P-values* < 0.05 are indicated as “*,” while comparisons not reaching statistical significance are not displayed.

### 3.3 Distinct Biophysical Properties of Vesicles Generated by PFA Plus DTT (Isotonic) versus PFA (Hypotonic) Methods

Given the known effects of DTT on proteins and lipids, we next investigated whether vesicles generated by the two methods under study differed in membrane organization and protein mobility. Lipid packing was assessed using a confocal microscopy-based GP measurement. C-Laurdan is highly sensitive to differences in the lipid order and can report these changes via altering their emission spectra.^30^ Hence, we performed GP measurements using C-Laurdan which revealed pronounced differences between the PMVs generated by the two protocols. PMVs produced using the PFA plus DTT (Isotonic) method exhibited significantly higher GP values, indicative of increased lipid order, compared to those generated by the PFA (Hypotonic) method (∼21% lower GP values; Figure 3A–C). To determine whether these changes can alter membrane protein dynamics, we measured the lateral mobility of CD1d receptor (labeled with Alexa Fluor 488-conjugated Fab antibody fragments) on vesicles derived from THP-1 CD1d cells via FCS. “*D*” values of CD1d were 2.1 ± 0.4 μm^2^/s for PMVs generated using PFA plus DTT (Isotonic) and 3.0 ± 0.75 μm^2^/s for those generated using PFA (Hypotonic) methods, corresponding to an approximately 30% reduction in the mobility of CD1d in PMVs generated in the presence of DTT (Mean ± SD; Figure 3D,E). These findings indicate that DTT would increase the membrane order and the two methods produce vesicles with distinct physicochemical properties.

**Figure 3:**
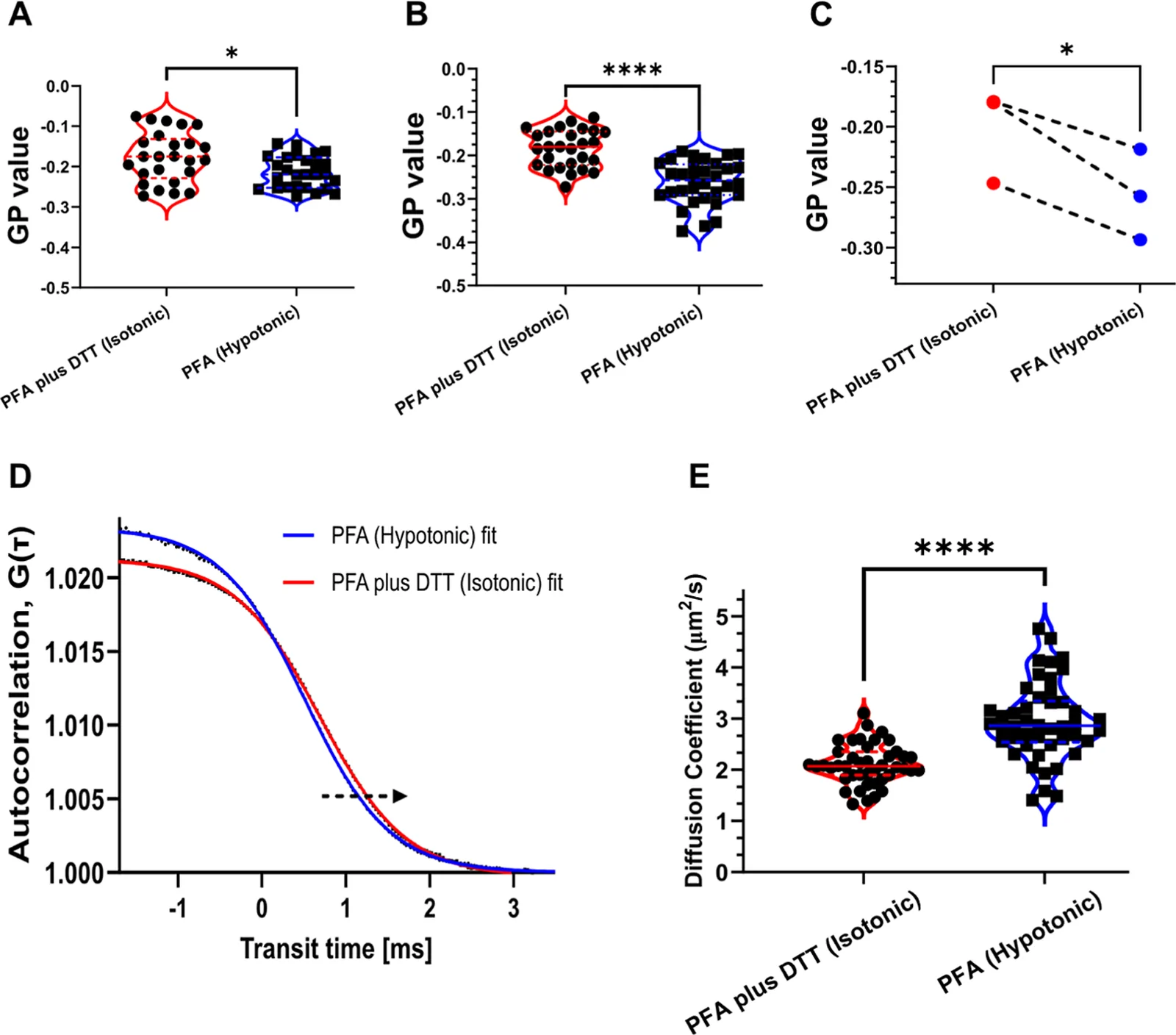
DTT significantly increases membrane lipid order decreasing the mobility of the transmembrane protein. PMVs were generated from THP1-CD1d cells with PFA plus DTT (Isotonic) and PFA (Hypotonic) methods. GP measurements of these PMVs were performed using C-Laurdan based on microscopy-based spectral imaging. (A, B) Data from independent experiments on different days. Each symbol represents data from a single PMV. (C) Comparison of median GP values from three independent experiments (*n* = 3). (D, E) Mobility measurements using FCS of the immune receptor CD1d on the top surface of PMVs. (D) Average autocorrelation curves from 42 FCS curves measured independently on separate PMVs, demonstrating slowing in the diffusion of CD1d in PFA plus DTT (Isotonic) condition. Red and blue lines are fits for the curves. (E) Diffusion coefficient (“D”) values from individual PMVs compared across both the conditions. In the figure, statistical analysis was performed using a two-tailed paired *t*-test with “*” and “****” indicating *p*-values < 0.05 and <0.0001, respectively.

### 3.4 Dialysis Renders PFA-Induced Vesicles Cytocompatible

Next, we assessed the cytocompatibility of PMVs using Jurkat iNKT cells for potential future biological applications. For this purpose, we focused exclusively on the PFA (Hypotonic) method since both methods contain similar amounts of PFA during the bleb-induction phase. Flow cytometry-based Annexin V/PI live–dead assays revealed that solution from control condition was nontoxic (Figure 4A–C,G,H), whereas solution from PFA (Hypotonic) condition was cytotoxic (Figure 4D–H). Hence, PMVs cannot be used right after their production. To eliminate cytotoxicity, we performed dialysis of the PMV-containing solution. Dialysis of solution from PFA (Hypotonic) condition resulted in gradual reduction of Jurkat iNKT cell death, with modest improvement after 3 h and near complete elimination of toxicity after 24 h of dialysis (Figure 4D–H). These results confirmed that dialysis is an effective strategy to remove residual chemicals, allowing PMVs to be used for subsequent biological experiments.

### 3.5 Antigen-Pulsed PMVs Effectively Induce T-Cell Activation

Once the cytocompatibility of PMVs was established, we next evaluated its functional performance in an immunologically relevant condition. Antigen presentation was selected as a model system, as it relies on close interactions between APCs and T cells. APCs express CD1d, which can present lipid antigen to iNKT cells, resulting in their activation. For this experiment, we used THP-1 CD1d cells as APC. THP-1 CD1d cells were cultured in the presence or absence of the lipid antigen α-GalCer. Next day, PMVs were generated from these cells and dialysis of the PMVs was performed for 24 h. Following dialysis, the PMV concentration was quantified using an Attune NxT flow cytometer. The PMVs were then cocultured with Jurkat iNKT cells at PMVs-to-cell ratios of 1:1 and 2:1 to assess their ability to induce antigen-specific T-cell activation. Jurkat iNKT cell activation was then assessed by determining the expression levels of CD69 and NF-κB-eGFP proteins. The data revealed that PMVs derived from α-GalCer-pulsed THP-1 CD1d cells were highly potent in inducing T-cell activation compared with control PMVs (Figure 5). Approximately, a 2-fold increase in the percentage of activated cells was observed based on the NF-κB-eGFP expression (Figure 5B,C). Also, activation of Jurkat iNKT cells was dependent on PMV doses with a higher concentration of PMVs inducing greater expression of NF-κB-eGFP (Figure 5D) and CD69 receptors (Figure 5E,F). These results demonstrate that PMVs faithfully reflect the physiological states of the cells of origin.

**Figure 4:**
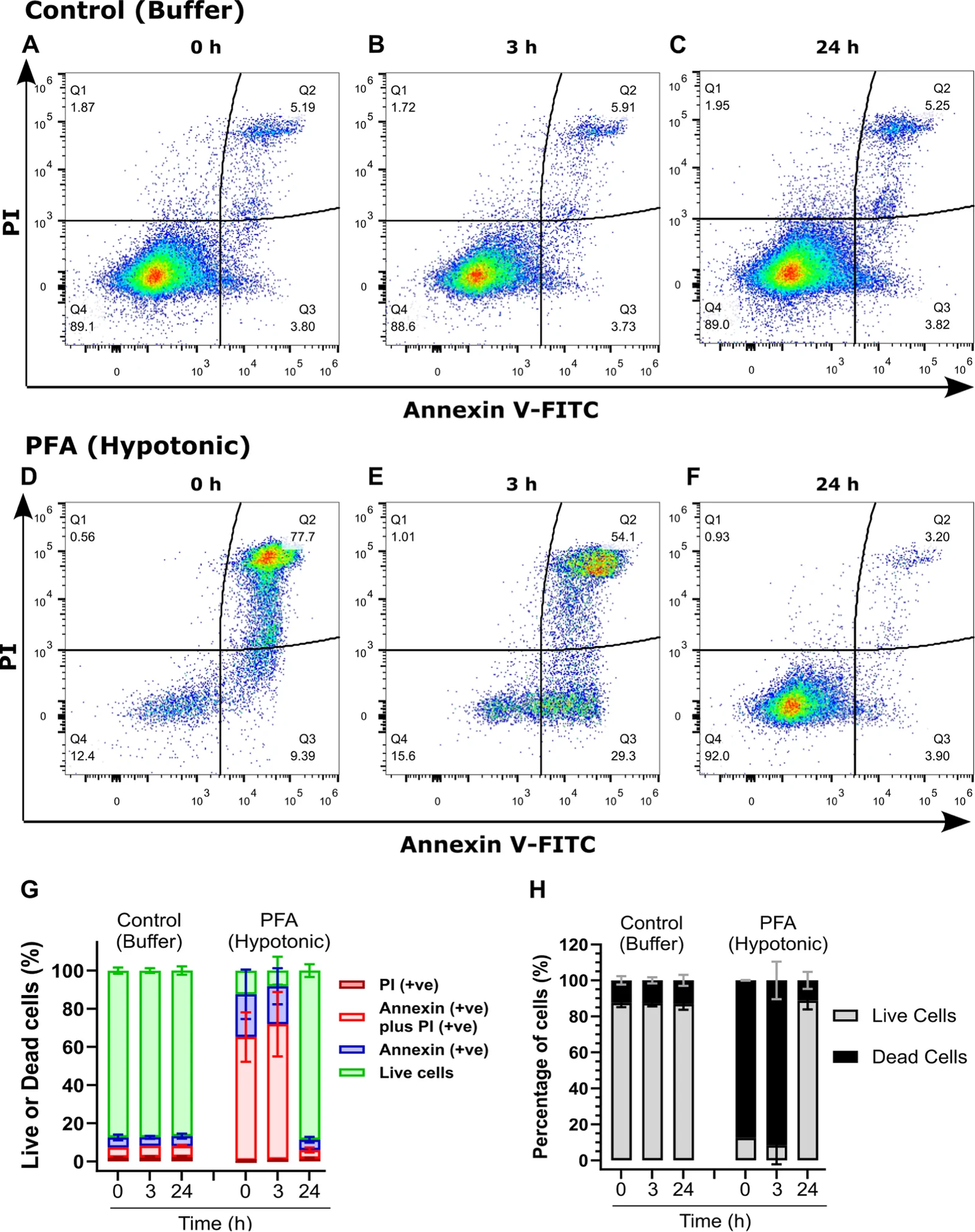
PFA is toxic to cells but dialysis post-PMV production makes it cytocompatible. PMVs were produced from THP1-CD1d cells and were processed as described in the materials and methods section. After the generation of PMVs, dialysis time-points were set as 0, 3, and 24 h. Subsequently, coculture experiments between PMVs and Jurkat iNKT cells were performed for 24 h. Thereafter, live–dead experiments were done by staining with Annexin V-FITC and PI. (A–F) 2D-scatter plots of Annexin V-FITC and PI: (A–C) Control and (D–F) PFA (Hypotonic). (G, H) Quantification of live and dead cells are shown: live cells are negative for both stains while dead cells are positive for either of the stains. (G) Percentage of Jurkat iNKT cells positive for either Annexin V-FITC or PI or both are demonstrated in grouped bar chart. (H) Cumulative dead cells vs live cells are shown. In the figure, data are presented as mean ± standard error of mean from two independent experiments (*n* = 2).

**Figure 5:**
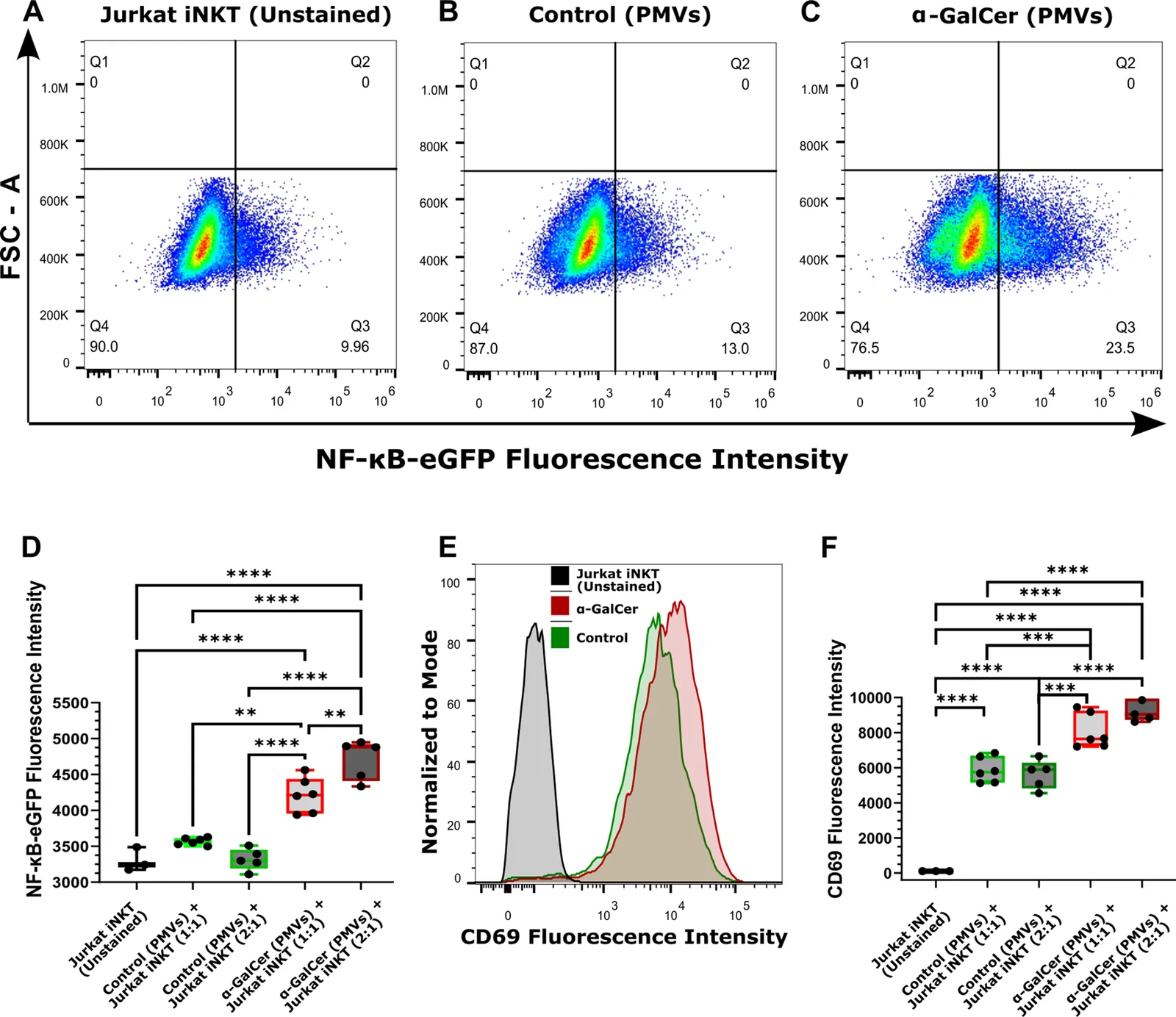
Antigen-presentation with PMVs. Coculture experiments were performed between PMVs and Jurkat iNKT cells. PMVs were produced from THP1-CD1d cells which were either cultured in the presence or absence of α-GalCer for 22 h: Control PMVs (buffer only) and α-GalCer PMVs (cultured with α-GalCer). PMVs from these two distinct conditions resemble distinct physiological states, i.e., activating or stimulatory and non-activating or nonstimulatory conditions. PMVs were dialyzed for 24 h and cocultured with Jurkat iNKT cells as described in the materials and methods section. They were then stained for the T-cell activation marker CD69 with the antibodies (APC conjugated, a fluorophore). (A–C) 2D-scatter plot of FSC-A vs NF-κB-eGFP fluorescence intentiy for Jurkat iNKT cells for three different conditions: (A) Unstained, (B) Control PMVs, nonstimulatory condition, and (C) α-GalCer PMVs, stimulatory condition. Comparison of median eGFP fluorescence intensity, NF-κB expression, among various groups is shown in (D), and for CD69 expression in (F). (E) Histogram of CD69 fluorescence intensity from Jurkat iNKT cells. The ratio of PMVs to Jurkat iNKT cells used in the coculture was 2:1. Assuming normal distribution of the data, one-way ANOVA followed by Tukey’s multiple comparison test was performed for statistical analysis. “**”, “***”, and “****” indicate *p*-values less than 0.01, 0.001, and 0.0001 respectively, while comparisons not reaching statistical significance are not shown in the figure. Experimental replicates performed on separate days are shown as independent symbol in the figure.

## 4 Discussion

PFA and DTT are the most widely used reagents for the generation of PMVs. While effective, DTT can significantly perturb membrane composition by reducing disulfide bonds and would promote endoplasmic reticulum stress through disruption of protein structure. In this study, we developed a DTT-free PMV generation method relying exclusively on PFA. A systematic comparison of this new approach with the conventional PFA plus DTT method revealed notable differences in vesicle yield, size, composition, and biophysical properties. Further, hypotonic treatment markedly enhanced vesiculation in the absence of DTT, yielding PMVs from both suspension and adherent cells with efficiency comparable to or exceeding that of the conventional method. PMVs generated using the PFA (Hypotonic) method were generally smaller and exhibited lower SSC values, suggesting reduced incorporation of cellular debris.

The most striking finding was the pronounced difference in the membrane lipid order. Elevated GP values in vesicles generated using PFA plus DTT indicate an increased membrane order, consistent with previous reports demonstrating that DTT alters the physicochemical properties of biological membranes. For example, DTT-induced endoplasmic reticulum stress has been shown to trigger lipid remodeling in yeast,^31^ and a recent study suggested loss of lipid asymmetry in GPMVs produced using PFA plus DTT.^12^ Furthermore, Levental et al. demonstrated that the altered physicochemical properties of GPMVs generated using the PFA plus DTT protocol arise from the combined action of both reagents, with DTT being the dominant factor affecting membrane order and phase-transition behavior, whereas PFA alone has only a minor influence on these properties.^10^ Our findings are consistent with these observations and further reveal functional consequences of DTT treatment, as reflected by the reduced lateral mobility of CD1d in the DTT-treated PMVs. Since mild PFA fixation has been shown to preserve native receptor properties, including palmitoylation,^7^ our results support the notion that PMVs generated using PFA alone are more likely to retain the native physicochemical and functional properties of the source-cell PM than PMVs isolated using the conventional PFA plus DTT protocol.

Importantly, we established that dialysis can effectively remove residual chemicals from PMV preparations, enabling their use in live cell assays. Using antigen presentation as a proof-of-principle, we showed that PMVs retain and transmit biologically relevant information reflective of the physiological state of the parent cells. Unlike living cells, PMVs lack the active cellular machinery required to remodel their membrane composition or reorganize membrane components during cellular interactions. Hence, they offer numerous advantages over cells, particularly in applications where controlled and stable membrane platforms are desirable. These properties, along with their amenability to modification and the potential to reduce their size to match extracellular vesicles (∼100 nm), make PMVs promising tools in mechanistic studies, drug delivery, and personalized therapeutic applications. In summary, this study introduces a robust, DTT-free PMV generation method that preserves key biological attributes of the PM, thereby expanding the utility of PMVs across both fundamental and translational research domains.

## 5 Conclusion

PMVs are widely used in the membrane biology research. However, DTT, a key reagent commonly used for PMV generation, can adversely affect protein function and PM organization. Consequently, PMVs generated using DTT may introduce experimental artifacts and may not accurately reflect the physiological state of the source cells. In this study, we developed a novel DTT-free method for producing PMVs by using PFA alone. We further demonstrate that DTT significantly alters PM organization by increasing the membrane order and reducing the membrane fluidity, thereby affecting the lateral mobility of the transmembrane receptor CD1d. We also established a simple dialysis-based purification strategy to remove residual vesiculating chemicals that are toxic to cells, thereby enabling the use of PMVs in biological assays. Using PMVs generated from APCs cultured in the presence or absence of antigen, we demonstrate that the new DTT-free method preserves distinct physiological states of the source cell PM, as evidenced by antigen-specific T-cell activation. Our findings highlight the limitations of DTT-based PMV production and establish a simple PFA-only strategy for generating PMVs that more faithfully preserve the native properties of the source cell PM. We anticipate that this approach will broaden the application of PMVs in biological research and further support their use as physiologically relevant model systems in both basic and translational research.

## Supplementary Material



## Notes

Flow cytometry gating strategy for identification and discrimination of PMVs and cells (PDF)

## §

National Heart and Lung Institute, Imperial College London, London W12 0NN, U.K

## ⊥

Department of Biophysical Imaging, Leibniz Institute of Photonic Technologies e.V., Albert-Einstein Strasse 9, 07745 Jena, Germany; member of the Leibniz Centre for Photonics in Infection Research (LPI), Jena, Germany; Institute of Applied Optics and Biophysics, Max- Wien Platz 1, 07743 Jena, Germany; Jena Center for Soft Matter (JCSM), Philosophenweg 7, 07743 Jena, Germany

## Abbreviations

PM: plasma membrane
Lo: liquid ordered
Ld: liquid disordered
PMVs: plasma membrane vesicles
DTT: dithiothreitol
PFA: paraformaldehyde
NEM: *N*-ethylmaleimide
CD1d: cluster of differentiation 1d
GP: generalized polarization
FCS: fluorescence correlation spectroscopy
Jurkat iNKT cell: jurkat invariant natural killer T cell
AO: acridine orange
FSC: forward scatter
SSC: side scatter
APC: antigen-presenting cell
α-GalCer: α-galactosylceramide
FWHM: full width at half-maximum
SD: standard deviation
