# Supplementary material for: Paraformaldehyde-Induced Vesiculation Preserves Native Membrane Physiological States Critical for T‑Cell Activation
Source: ACS Omega. 2026 Sep 3;11(37):55647–57. doi: 10.1021/acsomega.6c04767 (PMC13613478; doi:10.1021/acsomega.6c04767)
Supplement: Supplementary file 1 [file ao6c04767_si_001.pdf]

Paraformaldehyde-induced Vesiculation Preserves Native Membrane Physiological States Critical for T cell Activation

Dilip Shrestha,[*,†] Veerawut Veerapongchai, and Christian Eggeling[*,‡]

AUTHOR INFORMATION

Corresponding Authors

* **Dilip Shrestha** – *MRC Human Immunology Unit, Weatherall Institute of Molecular Medicine, University of Oxford, OX3 9DS, UK;*
https://orcid.org/0000-0002-6061-8350; Email: d.shrestha@imperial.ac.uk

* **Christian Eggeling** – *MRC Human Immunology Unit, Weatherall Institute of Molecular Medicine, University of Oxford, OX3 9DS, UK*;
https://orcid.org/0000-0002-3698-5599; Email: christian.eggeling@uni-jena.de

Authors

Veerawut Veerapongchai – *MRC Human Immunology Unit, Weatherall Institute of Molecular Medicine, University of Oxford, OX3 9DS, UK*

Present Addresses

[†] *(D.S.) National Heart and Lung Institute, Imperial College London, London W12 0NN, UK*

[‡] *(C.E.) Department of Biophysical Imaging, Leibniz Institute of Photonic Technologies e.V., Albert-Einstein Strasse 9, 07745 Jena, Germany; member of the Leibniz Centre for Photonics in Infection Research (LPI), Jena, Germany;Institute of Applied Optics and Biophysics, Max-Wien Platz 1, 07743 Jena, Germany; Jena Center for Soft Matter (JCSM), Philosophenweg 7, 07743 Jena, Germany*

Supplementary Figure

Figure S1.

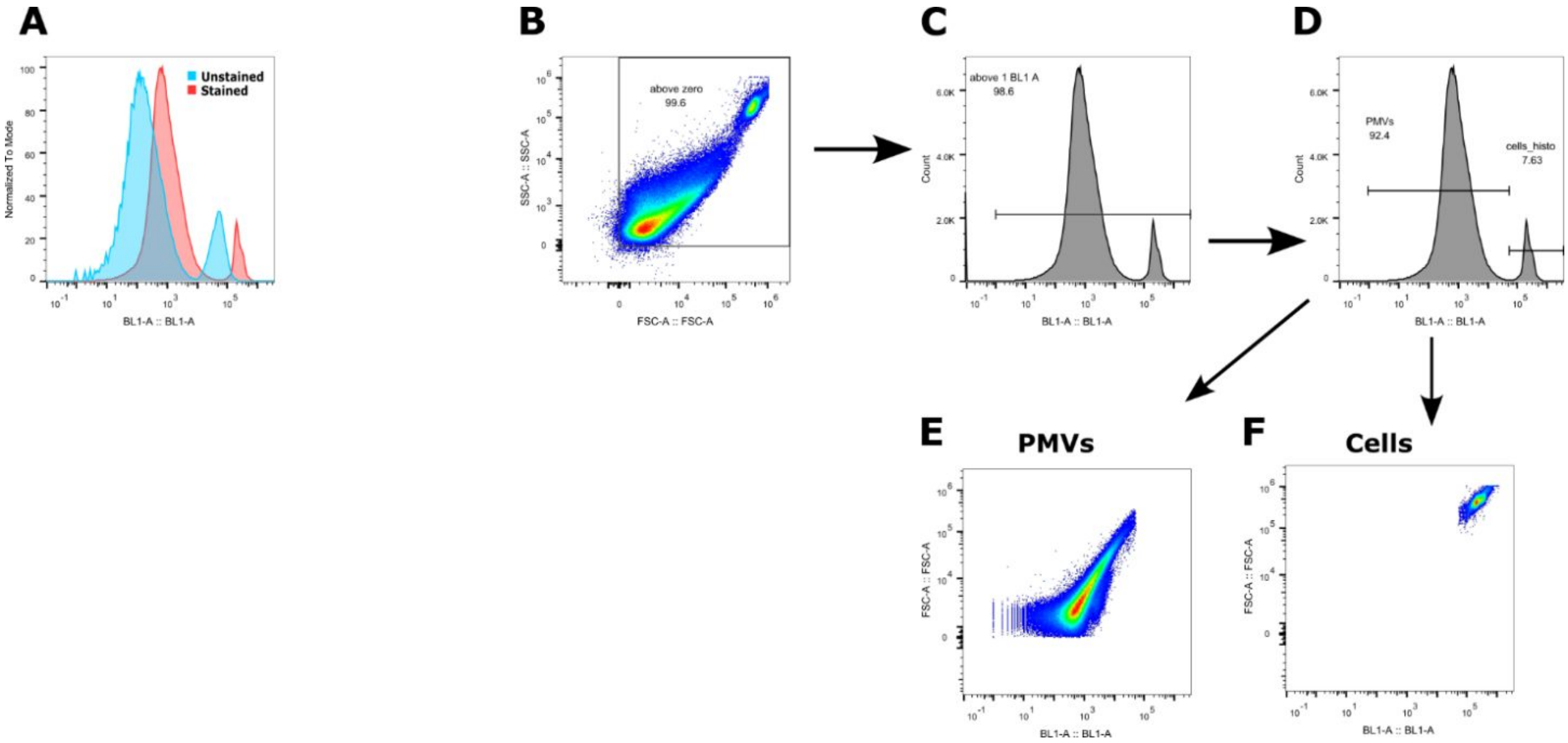

Figure S1. Flow cytometry gating strategy for identification and discrimination of PMVs and cells.

PMVs were generated using the PFA plus DTT (isotonic) method and analysed by flow cytometry. The sequential gating strategy used to identify PMVs and distinguish them from intact cells is shown. (A) Overlaid histograms of BL1-A fluorescence intensities are plotted for unstained and AO-stained samples. AO staining increases fluorescence signal intensity and improves discrimination of vesicles from background. (B) Dot plot of SSC-A (Y-axis) plotted against FSC-A (X-axis), showing the initial gate applied to include events with scattering values greater than 0, thereby selecting particles consistent with cells and PMVs. (C) Histogram of BL1-A fluorescence intensity (AO signal) used to define AO-positive events. A fluorescence threshold (>1) was applied to exclude background and retain membrane-containing events. (D) Histogram of BL1-A fluorescence intensity showing separation of two populations corresponding to PMVs (lower fluorescence intensity) and intact cells (higher fluorescence intensity). Distinct gates were drawn to separate these populations. Dot plot of FSC-A (Y-axis) plotted against BL1-A fluorescence (X-axis) for events gated in panel D: (E) PMVs and (F) cells. This gating strategy enables reliable identification and discrimination of PMVs from intact cells and background events.
